# The styloid process and the formation of sigmoid sinus diverticulum: is there a link?

**DOI:** 10.1016/j.bjorl.2019.12.006

**Published:** 2020-01-15

**Authors:** Zheng-Cai Lou

**Affiliations:** Yiwu Central Hospital, Department of Otorhinolaryngology, Yiwu, China

**Keywords:** Pulsatile tinnitus, Sigmoid sinus diverticulum, Internal jugular vein, Styloid process, Computed tomography venogram

## Abstract

**Introduction:**

Sigmoid sinus diverticulum has been considered the most common cause of pulsatile tinnitus; the mechanism underlying sigmoid sinus diverticulum formation is unclear. To the best of our knowledge, no previous studies have assessed whether the formation of sigmoid sinus diverticulum is related to compression of the internal jugular vein by the styloid process.

**Objective:**

To discuss the relationship between the styloid process and the formation of sigmoid sinus diverticulum.

**Methods:**

The medical records of nine patients diagnosed with venous pulsatile tinnitus caused by sigmoid sinus diverticulum were reviewed between April 2009 and May 2019. All patients underwent high-resolution computed tomography of the temporal bones, computed tomography venogram of the head and neck, magnetic resonance venography, and brain magnetic resonance imaging. The length and medial angulation of the styloid process were measured, and compression of the internal jugular vein was recorded.

**Results:**

The study population consisted of nine female right-sided pulsatile tinnitus patients with a mean age of 53.8 ± 4.6 years. The mean lengths of the styloid process were 3.9 ± 0.6 cm on the right side and 4.1 ± 0.7 cm on the left side. The mean medial angulation of the styloid process was significantly smaller on the right side than the left side (65.3° ± 1.2° vs. 67.8° ± 1.7°, *p* < 0.05). In addition, computed tomography venogram of the head and neck demonstrated the left internal jugular vein was compressed by the styloid process in eight of the nine patients.

**Conclusion:**

The formation of sigmoid sinus diverticulum with venous pulsatile tinnitus may be related to compression of the contralateral internal jugular vein by the styloid process. However, accumulation of data in additional cases is required to verify this suggestion.

## Introduction

Pulsatile tinnitus (PT) accounts for approximate l4 % of all cases of tinnitus.^1^, ^2^ Sigmoid sinus diverticulum (SSD) has been considered the most common cause of PT.^3^, ^4^, ^5^ However, there is ongoing debate regarding the origin of SSD. As most patients develop PT in late middle age, and therefore congenital SSD seems unlikely, SSD may be caused by flow forcefully hitting the sinus wall and thus leading to flow-induced outward remodeling and diverticulum formation.^6^ Previous studies have suggested that the majority of cases of PT and SSD involve the right side in females, and this may be associated with the right-sided dominance of the cerebral venous system.^7^, ^8^ In addition, clinical studies have shown that a few venous PT patients experience no relief or report recurrence after a single surgery.^4^, ^9^, ^10^ Dong et al.^11^ reported that the sound of venous PT may be triggered by multiple lesions within the veins.

Eagle^12^ first described the association between cervicofacial pain and other symptoms with an elongated styloid process (SP) in 1937. Eagle Syndrome is divided into two types, i.e., classic stylocarotid syndrome and stylocarotid artery syndrome (SAS),^13^, ^14^ and is more frequently seen on the right side if unilateral and is more common in females in late middle age.^12^, ^13^, ^14^ Therefore, the demographics of Eagle syndrome are consistent with those of SSD. SAS is essentially a vascular type of Eagle syndrome, which results in neurological symptoms, including transient ischemic attack and stroke, due to internal carotid artery (ICA) compression or dissection caused by an abnormal SP.^15^, ^16^, ^17^ To the best of our knowledge, no previous studies have assessed whether the internal jugular vein (IJV) is compressed similarly to compression of the ICA by an abnormal SP or whether the formation of SSD is related to compression of the IJV by the SP. This is the first study to retrospectively analyze the relationship between the formation of SSD and an abnormal SP.

## Methods

Ethical approval was provided by the Institutional Review Board of YiWu Central Hospital (Nº 20190416). Informed consent was obtained from all patients.

A retrospective review was performed among patients who presented to the YiWu Central Hospital with a chief complaint of unilateral persistent PT secondary to SSD based on radiographic evidence between February 2010 and March 2019. Each patient underwent full otoendoscopy, audiometric, and tympanometric evaluations, plus three-dimensional computed tomography (3DCT) of the temporal bones, computed tomography venography (CTV) of the head and neck, brain magnetic resonance imaging (MRI), and magnetic resonance venography (MRV). PT decreased transiently in response to compression of the ipsilateral cervical vascular structures but worsened following pressure release in all patients. SSD was defined as protrusion of the sinus wall into an aerated mastoid or into nonaerated cortex. Data on SSD and SP were obtained by searching radiology reports and images. The length and medial angulation of the SP (medial angle between the line crossing from the base of the SP and its trunk) were measured.^18^ Clinic and hospital charts, including operative reports, were examined retrospectively. Demographic information, including age, sex, duration of PT, and laterality of disease, were recorded for each patient. Mann-Whitney *U* test was used for comparison of the length and medial angulation degrees of both the sides.

## Results

**Figure 1:**
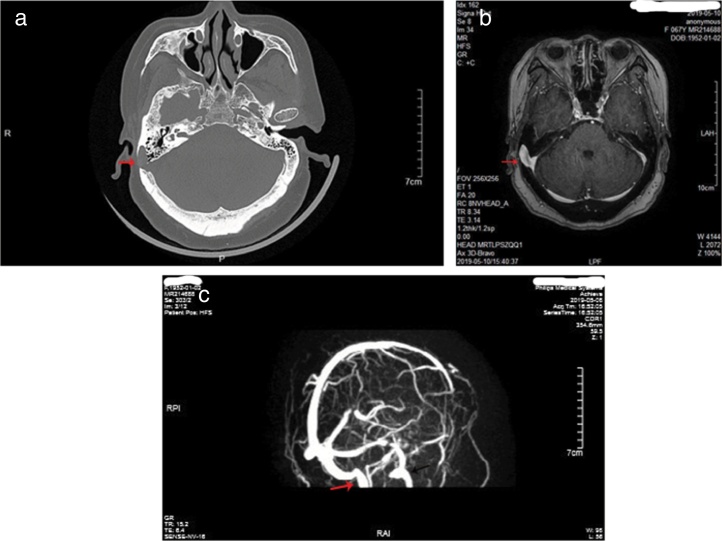
3DCT of the temporal bone revealed an intramastoid diverticulum originating from the right sigmoid sinus (a, red arrow). Enhanced MRI of the brain revealed intramastoid diverticulum of the right sigmoid sinus (b, red arrow). MRV revealed expansion of the sigmoid sinus, transverse sinus, and IJV on the right side compared to the left side (c, red arrow shows right IJV, black arrow shows left IJV).

**Figure 2:**
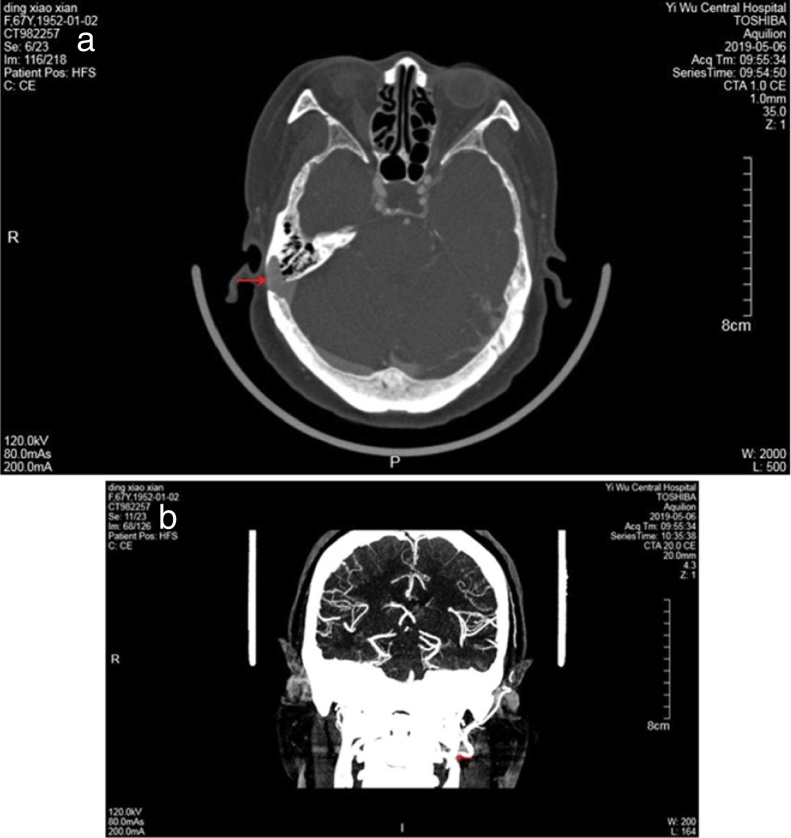
CTV revealed the diverticulum on the lateral surface of the right sigmoid sinus and a defect of the mastoid cortex (red arrow) (a). Compression of the left IJV by the SP (red arrow) (b).

The study population consisted of nine female patients with a mean age of 53.8 ± 4.6 years (range, 43–65 years), with a PT duration of 6.1 years (range, 1–12 years). All patients had right-sided PT and SSD, while none of idiopathic intracranial hypertension was found in 9 patients. Otoendoscopic examination revealed no middle ear abnormalities. The tinnitus was a crescendo-decrescendo objective low-frequency sound in 9 patients, which was consistent with the pulse in rhythm. The intensity decreased when the ipsilateral IJV was compressed. All the exams were performed in patients with pulsatile tinnitus, including audiometry, 3DCT, CTV of the head and neck, MRI, and MRV. Audiometry revealed mild bilateral, symmetrical, high-frequency sensorineural hearing loss in 5 patients, mild unilateral sensorineural hearing loss in the low frequencies in 2 patients, mixed hearing loss in one patient and normal hearing in one patient. 3DCT of the temporal bone and Enhanced MRI of the brain revealed intramastoid diverticulum of the right sigmoid sinus in 9 patients (Fig. 1). MRV revealed expansion of the sigmoid sinus, transverse sinus, and IJV on the right side compared to the left side (Fig. 1). CTV demonstrated diverticulum on the lateral surface of the right sigmoid sinus in 9 patients (Fig. 2).

The length and medial angulation of the SP are shown in Table 1. The mean length of the SP was 3.9 ± 0.6 cm for the right side and 4.1 ± 0.7 cm for the left side in the nine female patients. The mean medial angulation of the SP was significantly smaller on the right side than the left side (65.3° ± 1.2° vs. 67.8° ± 1.7°, respectively, *p* <  0.05). In addition, CTV of the head and neck demonstrated the left IJV was compressed by the SP in eight of the nine patients (Fig. 2); however, no IJV compression was found in the remaining patient. Only two patients occasionally experienced symptoms of head and neck pain, the feeling of a foreign body in the throat, and swallowing difficulty. Seven patients experienced no head and neck symptoms. Unfortunately, 5 patients with SSD refused surgery, of the 5 patients, 2 patients were afraid of surgery because of good times and bad of tinnitus, 2 patients asked for further observation because tinnitus had little effect on their life, and severe diabetes in one patient. Thus, sigmoid sinus wall reconstruction was performed in only four of the nine patients. Of these, the tinnitus disappeared after surgery in two patients, one patient felt relief from tinnitus, and the remaining patient experienced no change in PT after surgery.

**Table 1:** Lengths and medial angulation degrees of Styloid Process (SP) of 9 patients.

Case	Age	Sex	Styloid process		Medial angulation	
			Left side	Right side	Left side	Right side
1	65	F	3.0	2.2	64	69
2	54	F	2.8	4	67	71
3	57	F	5.3	3.9	66	66
4	46	F	5.2	4.5	68	65
5	48	F	4.8	3.2	65	67
6	52	F	3.9	5.1	63	68
7	61	F	3.7	3.8	66	70
8	59	F	4.2	4.6	64	69
9	43	F	4.1	4.1	65	66

## Discussion

SSD is a primary cause of venous PT, but its pathological mechanism remains unclear. A previous study showed that most cases of SSD occur on the right side in female patients.^1^, ^2^, ^3^, ^4^, ^5^, ^6^ Our observations are consistent with previous studies. SSD formation has been suggested to be attributable to right-sided venous dominance.^9^, ^19^, ^20^ In a radiographic study of the development of the sigmoid sinus, Friedmann et al.^20^ suggested that right-side dominance may be related to the normal nonsynchronous embryological development of venous sinuses leading to asymmetrical flow. Right-sided dominance of the sigmoid sinus means that an increased diameter of sigmoid sinus makes turbulent flow more likely than on the contralateral side.^20^, ^21^ It remains unclear whether variation in or compression of the contralateral IJV may affect the formation of SSD.

It is unclear whether SSD is related to compression of the contralateral IJV by the SP. Our observations indicated that SSD was consistent with Eagle Syndrome with regard to sex and the age of onset. All nine patients were female and ranged in age from 40 to 60 years old. Previous studies have suggested that SAS is mainly due to compression of the ICA by the SP; in this type, the SP is not necessarily elongated, but it can also be due to lateral or medial deviation.^3^, ^22^ In this study, although only two patients occasionally experienced symptoms of head and neck pain and the feeling of a foreign body in the throat, the left IJV was compressed by the SP in eight patients. The length of the SP was normal in eight patients, with only one patient showing elongation of the SP; however, the left medial angulation was larger than on the right side. Although compression of the ICA is more closely related to a decrease in medial angulation,^23^ we postulated that increased medial angulation may compress the IJV. Anatomically, the right-sided venous drainage system from the brain, measured at the level of the jugular foramen, is a dominant system in most cases; in particular, the volume of the right jugular foramen is significantly greater than that on the left only in females.^24^ This raises questions of why the right IJV and sigmoid sinus can easily become larger in these patients. We speculated that SSD formation may be related to compression of left IJV. Long-term compression of the left IJV resulted in obstruction of IJV reflux, increased backflow of blood on the right side, and abnormal expansion of the sigmoid sinus, transverse sinus, and jugular bulb, thereby leading to flow-induced outward remodeling and diverticulum formation. On the other hand, compression of the ICA by the SP may cause SAS and compression of the IJV may be asymptomatic because of a compensatory increase in contralateral IJV and the cerebral venous reflux system. Nevertheless, augmented or turbulent flow within the transverse–sigmoid–jugular venous system is one of the most common causes of venous PT.^25^ Certainly, variation in the IJV is also important for compression of the IJV by the SP. This may also explain why some patients with venous PT experience no relief or report recurrence after sigmoid sinus wall reconstruction. Although sigmoid sinus wall reconstruction could improve or repair the SSD, increased backflow of the ipsilateral IJV would cause focal vigorous turbulence of the blood flow, and thus sound energy from the turbulence may still be conducted to the middle ear via air or bone through adjacent bony structures or soft tissue. Therefore, it is crucial that radiologists identify every potential anomaly and variant of the venous system in preoperative CT and sufficiently describe these lesions to clinicians. This study was intended to remind clinicians that SSD formation could be related to an abnormal SP. However, further studies on larger populations are required to verify our results.

This study had limitations related to the small sample size and the absence of control subjects (i.e., age- and sex-matched patients without sigmoid sinus diverticuli or pulsatile tinnitus).

In addition, the lack of surgical treatment in most cases prevented confirmation that PT was related to the SSD and it was not possible to obtain direct evidence of the formation of SSD and SP. However, we conclude that 3DCT of the SP and CTV of the head and neck should be performed to exclude whether the IJV is compressed by the SP.

## Conclusions

Although SSD is the primary cause of venous PT, the mechanism underlying SSD formation is unclear. The formation of SSD may be related to compression of the contralateral IJV by the SP. However, radiographic evidence in a further multicenter study with a larger sample size is needed to confirm this suggestion.

## Conflicts of interest

The author declares no conflicts of interest.
